# Supplementary material for: In vitro-transcribed antigen receptor mRNA nanocarriers for transient expression in circulating T cells in vivo
Source: Nat Commun. 2020 Nov 27;11:6080. doi: 10.1038/s41467-020-19486-2 (PMC7695830; doi:10.1038/s41467-020-19486-2)
Supplement: Supplementary file 1 — Supplementary Information [file 41467_2020_19486_MOESM1_ESM.pdf]

Supplementary Information

In vitro-transcribed antigen receptor mRNA nanocarriers for transient expression in circulating T cells in vivo

Parayath, et al.

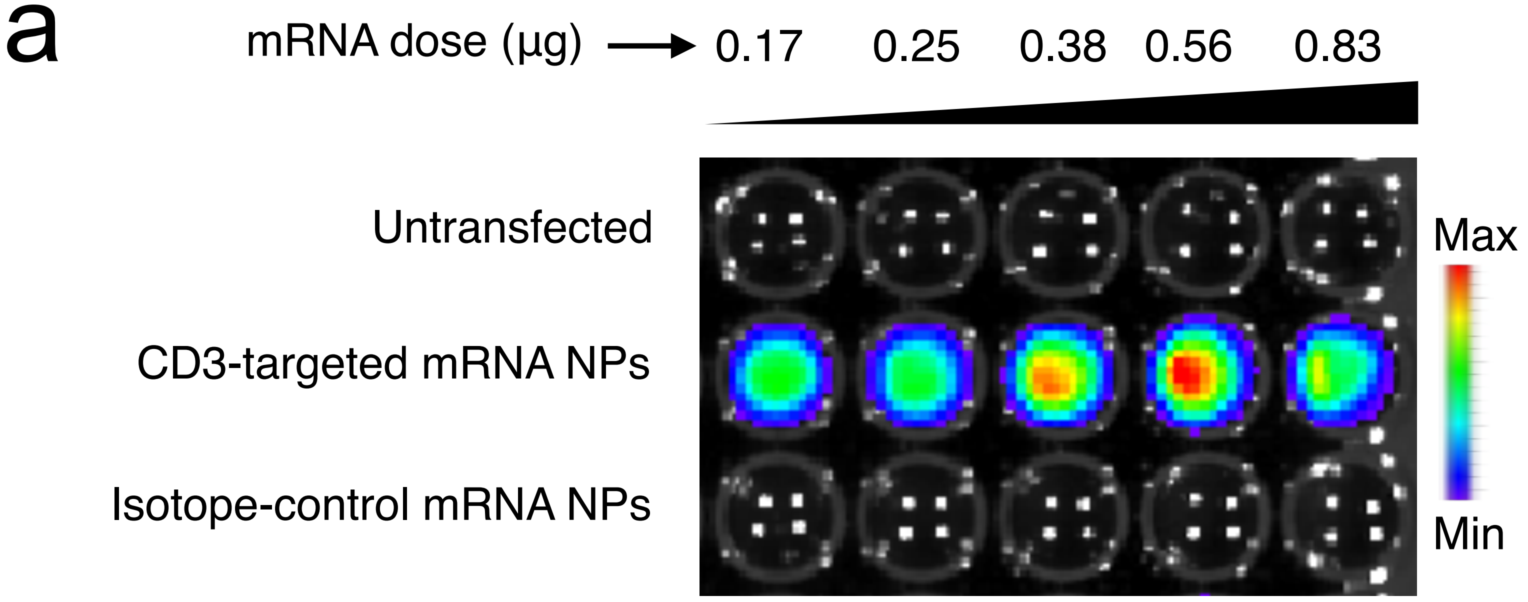

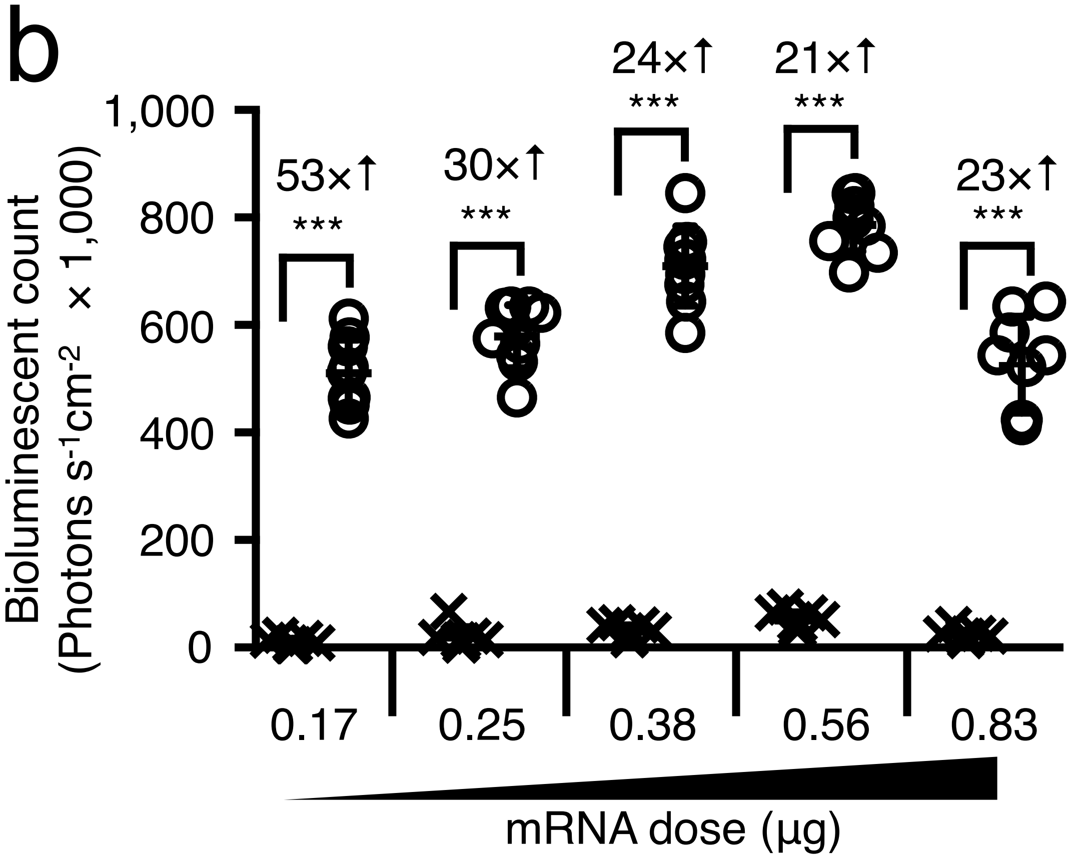

Supplementary Figure 1: Selective transfection of human T cells with CD3-targeted mRNA nanoparticles. PGA conjugates were formed with either anti-human CD3 (Bio X Cell, clone OKT3, cat#BE00001-2) or isotype control (Miltenyi mouse IgG2, clone S43.10, cat#130-106-546). Nanoparticles were formed by vortexing firefly luciferase mRNA, PBAE and PGA conjugate (1:60:2.5) in 25 mM NaOAc, pH 5.2. Nanoparticles were then directly added to purified human T cells at the indicated concentrations and luciferase expression was measured 24 hours later. (**a**) Bioluminescent signals emanating from transfected T cells. (**b**) Graphs showing mean photon counts. ***; significant, P<0.0001. N=9 biologically independent samples pooled from three independent experiments.

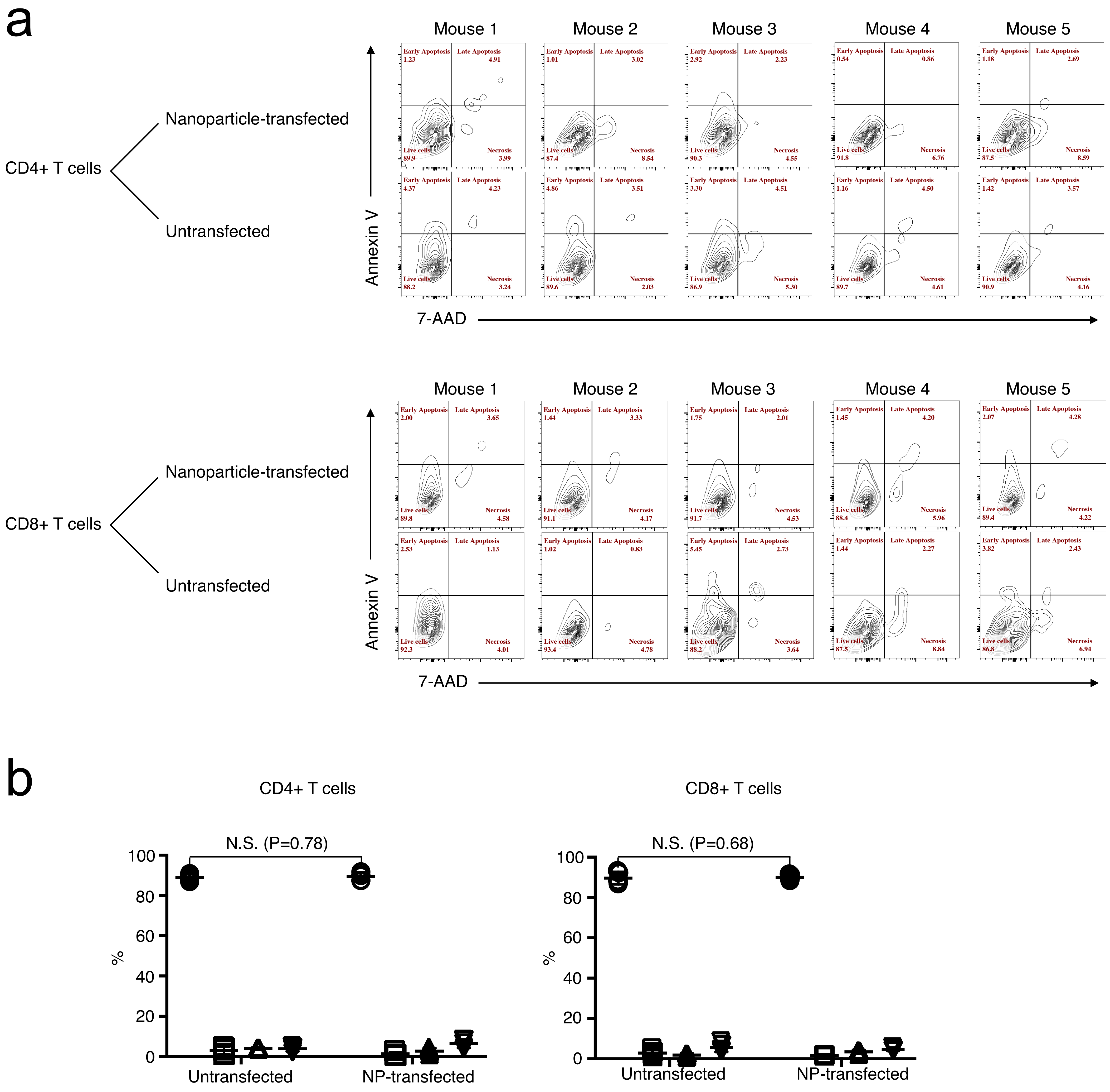

Supplementary Figure 2: *In situ* transfection of T cells does not compromise their viability. Ai14 reporter mice (n=5) were injected intravenously with three daily doses (day 1-3) of nanoparticles loaded with 15 µg mRNA encoding nuclear localization signal (NLS)-Cre. Nanoparticles were targeted to mouse T cells using a full-length anti-CD3 MuIgG2a designed as LALAPG variants to ablate Fc receptor binding and complement activation. Forty-eight hours after the final injection (day 5), single-cell suspensions of spleens were labeled with antibodies against CD45 and T cell markers (CD4+ or CD8+), washed twice with cold cell staining buffer (Biolegend catalog # 420201) and then resuspended in Annexin V binding buffer (Biolegend catalog # 422201) at a concentration of 1×10^6 cells/ml. The apoptosis/necrosis markers used were Annexin A5 (Biolegend catalog # 640953, 1:20) and 7-AAD (Biolegend catalog # 420404, 1:100). Data were collected using a BD FACSymphony analyzer running FACSDIVA software (Beckton Dickinson) and analyzed using FlowJo 10.0 software. (**a**) FACS profiles of Annexin V/7-AAD staining of splenocytes using the following gates:

<u>CD4+ T cells, nanoparticle-transfected:</u> CD45+ dtTomato+ CD4+
<u>CD4+ T cells, untransfected:</u> CD45+ dtTomato- CD4+
<u>CD8+ T cells, nanoparticle-transfected:</u> CD45+ dtTomato+ CD8+
<u>CD8+ T cells, untransfected:</u> CD45+ dtTomato- CD8+

(**b**) Graphs displaying the mean % ± SD of live, early apoptotic, late apoptotic, and necrotic cells. Pairwise differences were statistically analyzed by two-tailed Student's t-test. N=5 biologically independent samples.

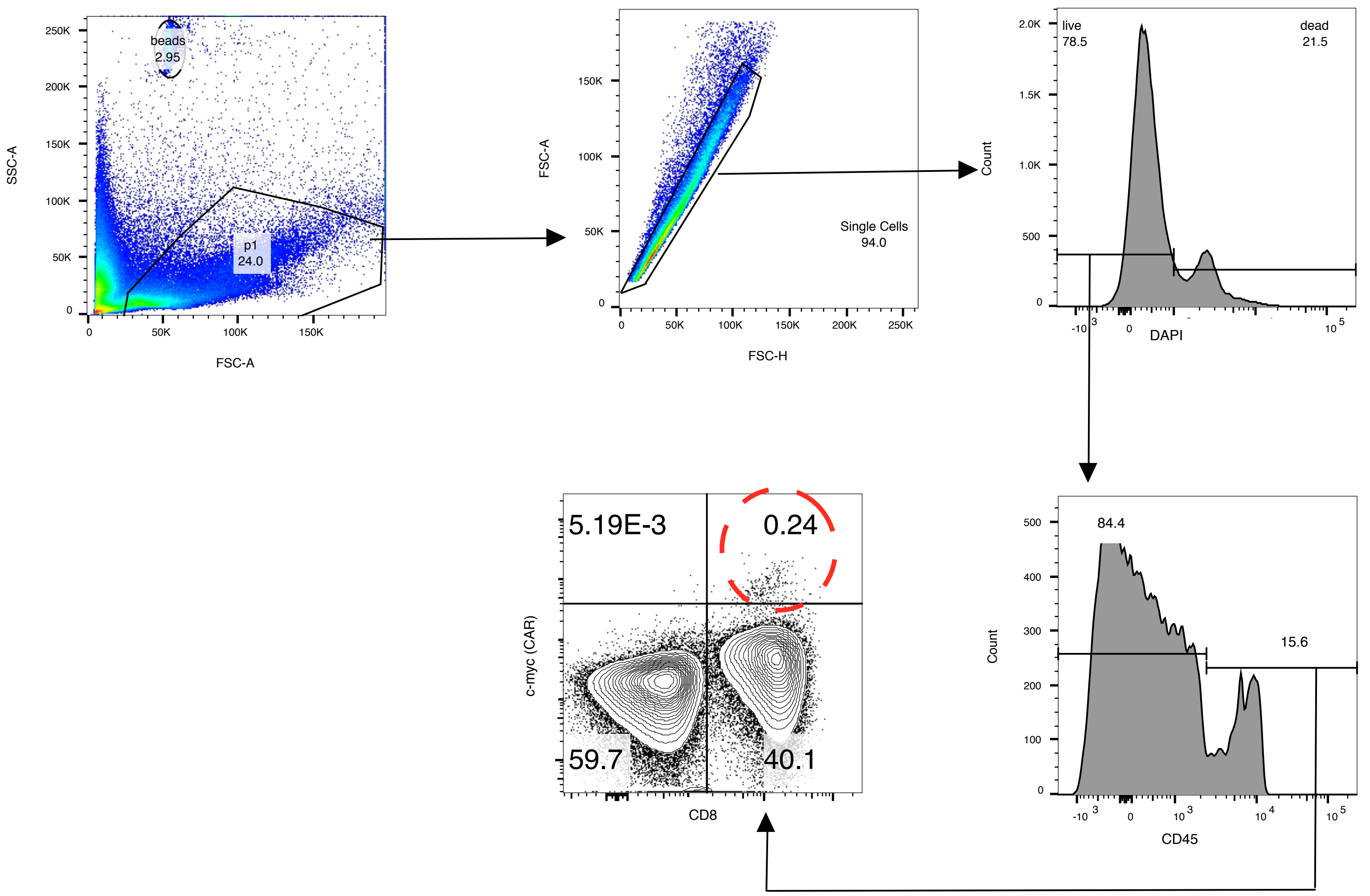

Supplementary Figure 3: Gating strategy for multicolor flow cytometry analysis shown in Figure 4d. Peripheral blood was stained with antibodies against cell surface markers as indicated and followed with flow cytometry (see methods). Cells were first gated on size and singularity followed by DAPI exclusion to identify live cells for further analysis. Live cells were gated on CD45 before identifying CD8+ c-myc+ (CAR+) cells.

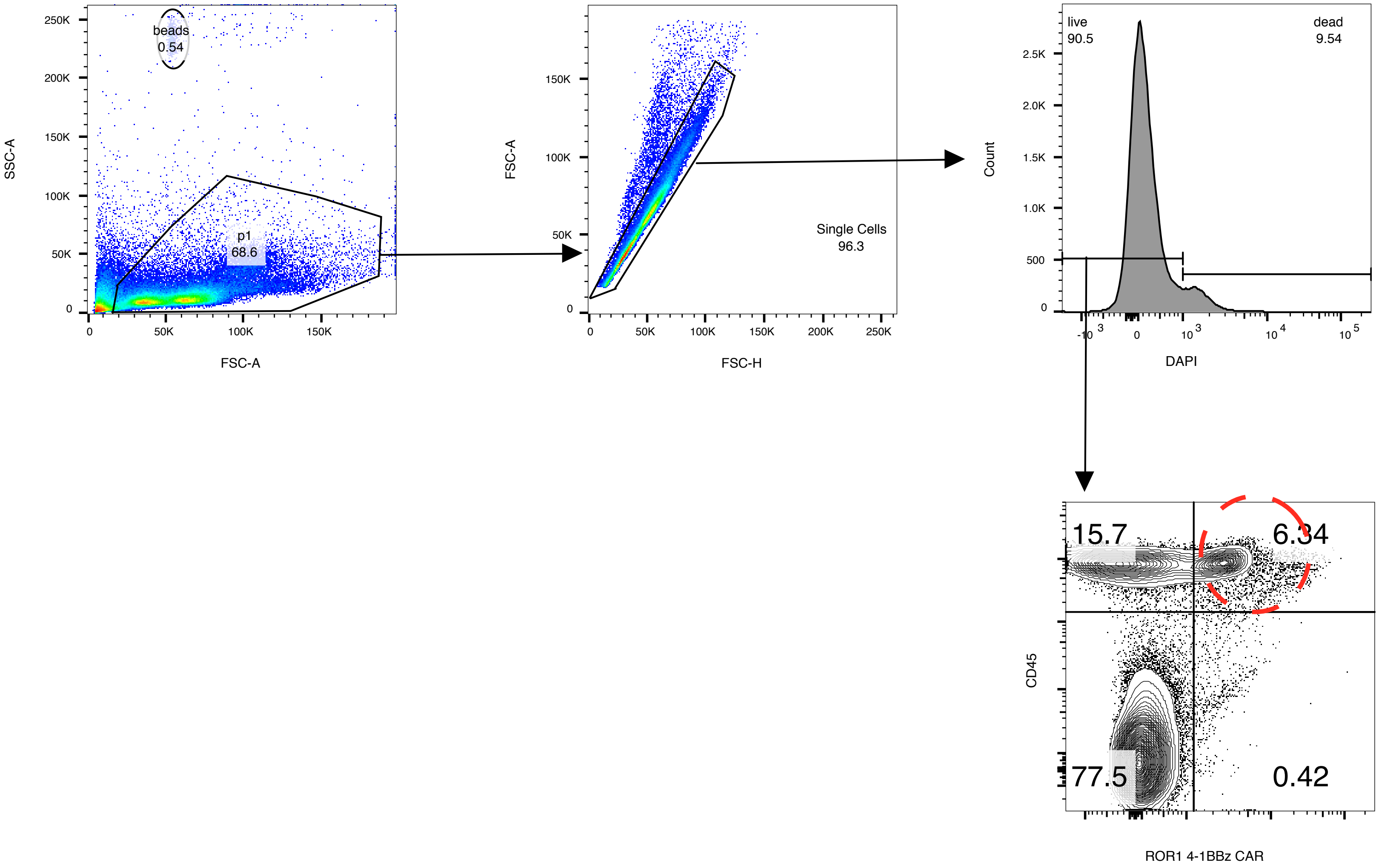

Supplementary Figure 4: Gating strategy for multicolor flow cytometry analysis shown in Figure 7g. Tumors were digest and single cell suspensions were stained with antibodies against cell surface markers as indicated and followed with flow cytometry (see methods). Cells were first gated on size and singularity followed by DAPI exclusion to identify live cells before identifying CD45+ROR1 4-1BBz CAR+ cells.

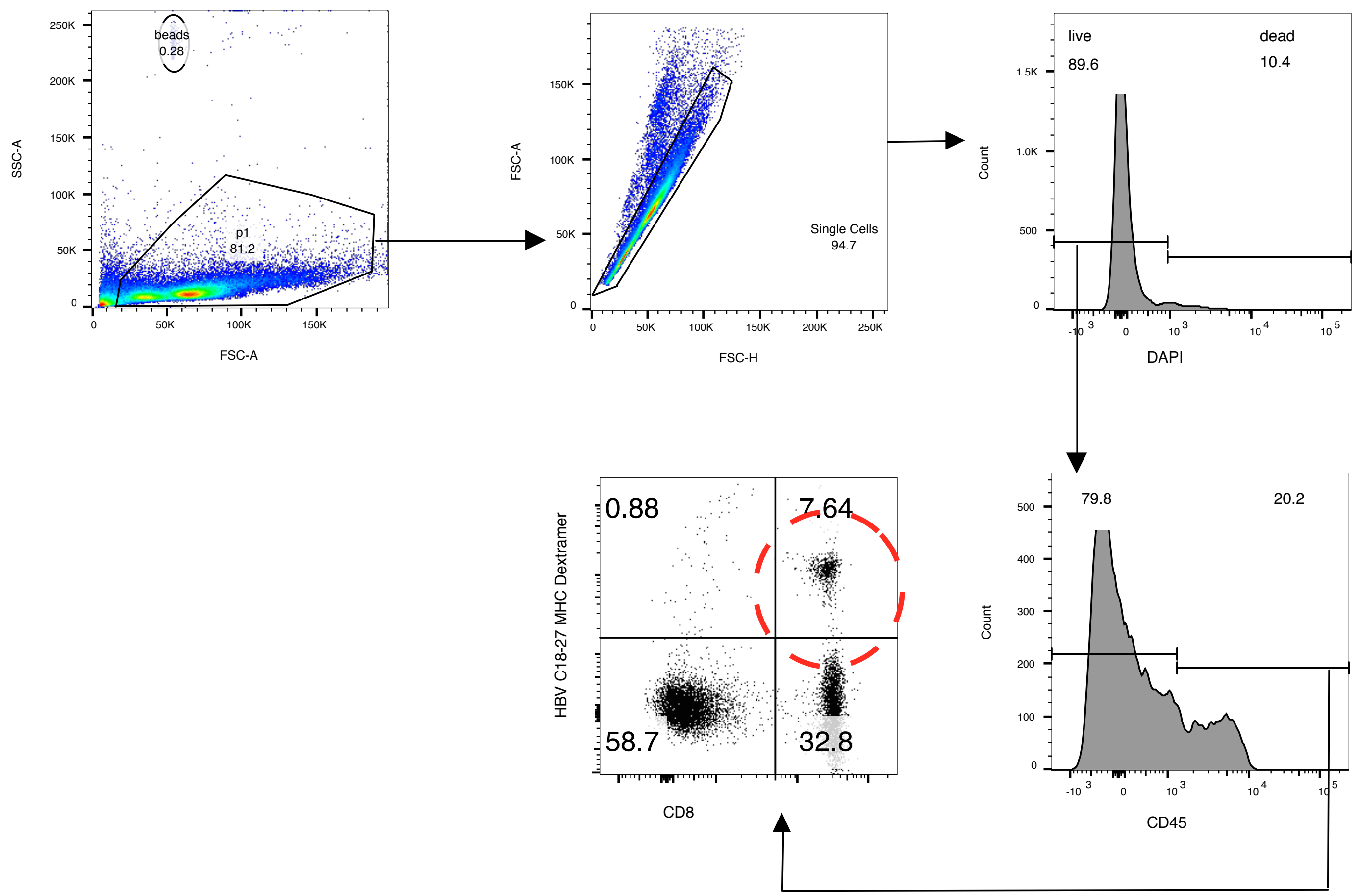

Supplementary Figure 5: Gating strategy for multicolor flow cytometry analysis shown in Figure 8d. Livers were digest and single cell suspensions were stained with antibodies against cell surface markers as indicated and followed with flow cytometry (see methods). Cells were first gated on size and singularity followed by DAPI exclusion to identify live cells for further analysis. Live cells were gated on CD45 before identifying CD8+ HBV C18-27 MHC Dextramer+ cells.

Supplementary Table 1 List of primary antibodies and other staining reagents used for flow cytometry

Specificity	Clone	Isotype	Dilution	Dye	Supplier	Catalog#
Human CD8	3B5	IgG2a	1:400	APC-Alexa Fluor 750	ThermoFisher	MHCD0827
Human CD3	UCHT1	IgG1, kappa	1:400	Alexa Fluor 532	ThermoFisher	58-0038-42
Human CD45	HI130	IgG2a	1:400	PE-Cyanine 7	ThermoFisher	25-0459-42
c-Myc	9B11	IgG2a	1:50		Cellsignal.com	3739S
Human ROR-1		IgG	1:200	Donkey anti-Goat IgG secondary antibody, Alexa Fluor Plus 647 (ThermoFisher, Cat# A32849)	Antibodies-online.com	ABIN4899817
PSMA		IgG	1:300	FITC	LifeSpan BioSciences	LS-C317473-50
Human PSCA	7F5	IgG2b kappa	1:200	PE	Santa Cruz Biotechnology	Sc-80654
HLA-A201-HBV core 18-27-PE pentamer	-	-	1:200	PE	Proimmune	F023-2B - 23 - A*02:01 - FLPSDFFPSV - **Pentamer** - 150 test R-PE
Live/Dead Fixable Green	-	-	1:800	FITC	Life Technologies	L23101
Mouse CD45	30-F11	IgG2b, κ	1:100	BUV661	BD Biosciences	612975
Mouse CD11b	M1/70	IgG2b, κ	1:100	BUV395	BD Biosciences	563553
Mouse CD11c	HL3	IgG1, λ2	1:100	APC-Cy™7	BD Biosciences	561241
Mouse LY6G	1A8	IgG2a, κ	1:100	BUV737	BD Biosciences	741813
Mouse CD19	1D3	IgG2a, κ	1:100	BUV805	BD Biosciences	749027

Mouse F4/80	BM8	IgG2a, κ	1:200	FITC	Biolegend	123108
Mouse CD4	RM4-5	IgG2a, κ	1:100	Brilliant Violet 421	Biolegend	100563
Mouse CD8a	53-6.7	IgG2a, κ	1:50	PerCP/Cyanine5.5	Biolegend	100734
Mouse CD44	IM7	IgG2b, κ	1:100	BUV496	BD Biosciences	741057
Mouse CD62L	MEL-14	IgG2a, κ	1:50	BV650	Biolegend	104453
Mouse CD69	H1.2F3	IgG1, λ3	1:100	BUV563	BD Biosciences	741234
Live/dead stain - Zombie Aqua	-	-	1:400	BV510	Biolegend	423101
Annexin A5	-	-	1:20	APC/Fire™ 750	Biolegend	640953
7-AAD	-	-	1:100	-	Biolegend	420404

Supplementary Methods

Anti-CD3 murine IgG2a LALA-PG and nonbinder control (used only for in vivo experiments in immunocompetent mice/ Figure 6)

Heavy Chain: anti-CD3 MuIgG2a LALA-PG:

MELGLCWVFLVAILEGVQCEVKLVESGGGLVQPGKSLKLSCEASGFTFSGYGMHWVRQAPGRGLESVAYITSSSINIKYLDAVKGRF

TVSRDNAKNLLFLQMNILKSEDTAMYYCARFDWDKNYWGQGTMVTVSSAKTTAPSVYPLAPVCGDTTGSSVTLGCLVKGYFPEPVTL

TWNSGSLSSGVHTFPAVLQSDLYTLSSSVTVTSSTWPSQSITCNVAHPASSTKVDKKI**EPRGPTIKPCPPCKCGAPNAAGGPSVFIFPP**

KIKDVLMISLSPIVTCVVVDVSEDDPDVQISWFVNNVEVHTAQTQTHREDYNSTLRVVSALPIQHQDWMSGKEFKCKVNNKDLP

APIERTISKPKGSVRAPQVYVLPPPEEEMTKKQVTLTCMVTDFMPEDIYVEWTNNGKTELNYKNTEPVLDSDGSYFMYSKLRVE

KKNWVERNSYSCSVVHEGLHNHHTTKSFSRTPG

Light Chain: anti-CD3 murine constant Kappa (VK):

MDMRVPAQLLGLLLLWLPGAKCDIQMTQSPSSLPASLGDRVTINCQASQDISNYLNWYQQKPGKAPKLLIYYTNKLADGVPSRFSGS

GSGRDSSFTISSLESEDIGSYYCQQYYNYPWTFGPGTKLEIKRADAAPTVSIFPPSSEQLTSGGASVVCFLNNFYPKDINVKWKIDGSERQ

NGVLNSWTDQDSKDSTYSMSSTLTLTKDEYERHNSYTCEATHKTSTSPIVKSFNRNEC

As a specificity control, the following 2C11-based nonbinder protein was designed:

Nonbinder Heavy Chain with LALA-PG:

MELGLCWVFLVAILEGVQCEVKLVESGGGLVQPGKSLKLSCEASGFTFSGYGMHWVRQAPGRGLESVAYITSSSINIKYLDAVKGRF

TVSRDNAKNLLFLQMNILKSEDTAMYYCARGDSDGNSGGQGTMVTVSSAKTTAPSVYPLAPVCGDTTGSSVTLGCLVKGYFPEPVTLT

WNSGSLSSGVHTFPAVLQSDLYTLSSSVTVTSSTWPSQSITCNVAHPASSTKVDKKI**EPRGPTIKPCPPCKCGAPNAAGGPSVFIFPPKI**

KDVLMISLSPIVTCVVVDVSEDDPDVQISWFVNNVEVHTAQTQTHREDYNSTLRVVSALPIQHQDWMSGKEFKCKVNNKDLPAP

IERTISKPKGSVRAPQVYVLPPPEEEMTKKQVTLTCMVTDFMPEDIYVEWTNNGKTELNYKNTEPVLDSDGSYFMYSKLRVEK

KNWVERNSYSCSVVHEGLHNHHTTKSFSRTPGK

Nonbinder light kappa chain variable chain region (VK):

MDMRVPAQLLGLLLLWLPGAKCDIQMTQSPSSLPASLGDRVTINCQASQDISNYLNWYQQKPGKAPKLLIYYTNKLADGVPSRFSGS

GSGRDSSFTISSLESEDIGSYYCQQGSNGPWTFGPGTKLEIKRADAAPTVSIFPPSSEQLTSGGASVVCFLNNFYPKDINVKWKIDGSERQ

NGVLNSWTDQDSKDSTYSMSSTLTLTKDEYERHNSYTCEATHKTSTSPIVKSFNRNEC

Both proteins were expressed and purified by ATUM Biosciences (Newark, California, USA).
