# Supplementary material for: In vitro-transcribed antigen receptor mRNA nanocarriers for transient expression in circulating T cells in vivo
Source: Nat Commun. 2020 Nov 27;11:6080. doi: 10.1038/s41467-020-19486-2 (PMC7695830; doi:10.1038/s41467-020-19486-2)
Supplement: Supplementary file 3 — Reporting Summary [file 41467_2020_19486_MOESM3_ESM.pdf]

nature research

Corresponding author(s): Stephan MT
Last updated by author(s): Oct 13, 2020

Reporting Summary

Nature Research wishes to improve the reproducibility of the work that we publish. This form provides structure for consistency and transparency in reporting. For further information on Nature Research policies, see our Editorial Policies and the Editorial Policy Checklist.

Statistics

For all statistical analyses, confirm that the following items are present in the figure legend, table legend, main text, or Methods section.

n/a	Confirmed	
☐	☒	The exact sample size (n) for each experimental group/condition, given as a discrete number and unit of measurement
☐	☒	A statement on whether measurements were taken from distinct samples or whether the same sample was measured repeatedly
☐	☒	The statistical test(s) used AND whether they are one- or two-sided<br>*Only common tests should be described solely by name; describe more complex techniques in the Methods section.*
☐	☒	A description of all covariates tested
☒	☐	A description of any assumptions or corrections, such as tests of normality and adjustment for multiple comparisons
☐	☒	A full description of the statistical parameters including central tendency (e.g. means) or other basic estimates (e.g. regression coefficient) AND variation (e.g. standard deviation) or associated estimates of uncertainty (e.g. confidence intervals)
☐	☒	For null hypothesis testing, the test statistic (e.g. F, t, r) with confidence intervals, effect sizes, degrees of freedom and P value noted<br>*Give P values as exact values whenever suitable.*
☒	☐	For Bayesian analysis, information on the choice of priors and Markov chain Monte Carlo settings
☒	☐	For hierarchical and complex designs, identification of the appropriate level for tests and full reporting of outcomes
☒	☐	Estimates of effect sizes (e.g. Cohen's d, Pearson's r), indicating how they were calculated

Our web collection on statistics for biologists contains articles on many of the points above.

Software and code

Policy information about availability of computer code

Data collection	Bioluminescence was acquired using Living Image (R) version 4.3.1 software (Caliper Life Sciences). Flow cytometry data was acquired using FACSCanto software (FACSDiva v.6.1). Incucyte(R) S3 software (v2018B) was used to acquire data with the IncuCyte(R) Live Cell Analysis System.
Data analysis	Bioluminescence was analyzed using Living Image (R) version 4.3.1 software (Caliper Life Sciences). Statistical analysis was done with Prism software Version 7.0b (Graphpad). Flow cytometry data was analyzed with FlowJo version 10.3. Incucyte(R) S3 software (v2018B) was used to analyze data acquired with the IncuCyte(R) Live Cell Analysis System

For manuscripts utilizing custom algorithms or software that are central to the research but not yet described in published literature, software must be made available to editors and reviewers. We strongly encourage code deposition in a community repository (e.g. GitHub). See the Nature Research guidelines for submitting code & software for further information.

Data

Policy information about availability of data

All manuscripts must include a data availability statement. This statement should provide the following information, where applicable:

- Accession codes, unique identifiers, or web links for publicly available datasets
- A list of figures that have associated raw data
- A description of any restrictions on data availability

All data supporting the findings of this study are available within the article and its supplementary information files and directly from M. Stephan upon reasonable request. A reporting summary for this article is available as a Supplementary Information file.

Field-specific reporting

Please select the one below that is the best fit for your research. If you are not sure, read the appropriate sections before making your selection.

- [x] Life sciences
- [ ] Behavioural & social sciences
- [ ] Ecological, evolutionary & environmental sciences

For a reference copy of the document with all sections, see nature.com/documents/nr-reporting-summary-flat.pdf

Life sciences study design

All studies must disclose on these points even when the disclosure is negative.

Sample size	We carried out our tumor studies in 10 mice per treatment group. This sample size provided us with 90% power to detect an effect size of 1 SD between groups, based on a one-way analysis of variance (ANOVA) with 2-sided significance level of 0.05 (calculated with Prism 6.0 Graph Pad software). For in vitro assays, assay protocols were developed using single donors, then replicated with 3+ independent donors.
Data exclusions	All data were included in the analysis. No data were excluded from the analysis
Replication	All experiments were replicated at least two times by two independent scientists working as a team on this project. Bioluminescent imaging and flow cytometry data were acquired and analyzed by two postdoctoral fellows working on this project. All replication attempts were successful.
Randomization	Once tumors established, we randomized mice each into the control or treatment groups.
Blinding	Investigators were not blinded, since the key therapeutic readout was bioluminescent tumor imaging, which is an unbiased readout. All toxicity and safety studies (Figure 10) were conducted by a board-certified staff pathologist in a double-blinded fashion.

Reporting for specific materials, systems and methods

We require information from authors about some types of materials, experimental systems and methods used in many studies. Here, indicate whether each material, system or method listed is relevant to your study. If you are not sure if a list item applies to your research, read the appropriate section before selecting a response.

Materials & experimental systems

n/a	Involved in the study	
[ ]	[x]	Antibodies
[ ]	[x]	Eukaryotic cell lines
[x]	[ ]	Palaeontology and archaeology
[ ]	[x]	Animals and other organisms
[ ]	[x]	Human research participants
[x]	[ ]	Clinical data
[x]	[ ]	Dual use research of concern

Methods

n/a	Involved in the study	
[x]	[ ]	ChIP-seq
[ ]	[x]	Flow cytometry
[x]	[ ]	MRI-based neuroimaging

Antibodies

Antibodies used

Anti-human CD8 antibody for targeting nanoparticles to T lymphocytes:

Human CD8 (clone OKT8)
BioXcell (Cat#BE0004-2)

Flow cytometry antibodies:

Human CD8
3B5
IgG2a
1:400
APC-Alexa Fluor 750
ThermoFisher
MHCD0827

Human CD3

UCHT1
IgG1, kappa
1:400
Alexa Fluor 532
ThermoFisher
58-0038-42

Human CD45
HI130
IgG2a
1:400
PE-Cyanine 7
ThermoFisher
25-0459-42

c-Myc
9B11
IgG2a
1:50
Cellsignal.com
3739S

Human ROR-1
IgG
1:200
Donkey anti-Goat IgG secondary antibody, Alexa Fluor Plus 647 (ThermoFisher, Cat# A32849)
Antibodies-online.com
ABIN4899817

PSMA
IgG
1:300
FITC
LifeSpan BioSciences
LS-C317473-50

Human PSCA
7F5
IgG2b kappa
1:200
PE
Santa Cruz Biotechnology
Sc-80654

HLA-A201-HBV core 18-27-PE pentamer
1:200
PE
Proimmune
F023-2B - 23 - A*02:01 - FLPSDFFPSV - Pentamer - 150 test R-PE

Live/Dead Fixable Green
1:800
FITC
Life Technologies
L23101

Mouse CD45
30-F11
IgG2b, κ
1:100
BUV661
BD Biosciences
612975

Mouse CD11b
M1/70
IgG2b, κ

1:100
BUV395
BD Biosciences
563553

Mouse CD11c
HL3
IgG1, λ2
1:100
APC-Cy™7
BD Biosciences
561241

Mouse LY6G
1A8
IgG2a, κ
1:100
BUV737
BD Biosciences
741813

Mouse CD19
1D3
IgG2a, κ
1:100
BUV805
BD Biosciences
749027

Mouse F4/80
BM8
IgG2a, κ
1:200
FITC
Biolegend
123108

Mouse CD4
RM4-5
IgG2a, κ
1:100
Brilliant Violet 421
Biolegend
100563

Mouse CD8a
53-6.7
IgG2a, κ
1:50
PerCP/Cyanine5.5
Biolegend
100734

Mouse CD44
IM7
IgG2b, κ
1:100
BUV496
BD Biosciences
741057

Mouse CD62L
MEL-14
IgG2a, κ
1:50
BV650
Biolegend
104453

Mouse CD69
H1.2F3
IgG1, λ3
1:100
BUV563
BD Biosciences
741234

Live/dead stain - Zombie Aqua
1:400
BV510
Biolegend
423101

Annexin A5
1:20
APC/Fire™ 750
Biolegend
640953

7-AAD
1:100
Biolegend
420404

Validation

These commercially available flow cytometry antibodies have not been further validated by our laboratory.
Certificates of Analysis can be found here:

Human CD8 (ThermoFisher): https://assets.thermofisher.com/TFS-Assets/LSG/certificate/Certificates-of-Analysis/MHCD0827%202150638.pdf

Human CD3 (ThermoFisher): https://www.thermofisher.com/antibody/product/CD3-Antibody-Monoclonal/58-0038-42

Human CD45 (ThermoFisher): https://www.thermofisher.com/antibody/product/CD45-Antibody-Monoclonal/25-0459-42

c-myc (Cellsignal.com): https://media.cellsignal.com/coa/2276/24/2276-lot-24-coa.pdf

Human ROR-1 (Antibodies-online.com): https://www.antibodies-online.com/productsheets/ABIN4899817.pdf

PSMA (LifeSpan BioSciences): https://www.lsbio.com/antibodies/folh1-antibody-psma-antibody-fitc-elisa-ihc-wb-western-ls-c317473/327657#validation-section

PSCA (Santa Cruz): https://datasheets.scbt.com/sc-80654.pdf

HLA-A201-HBV core 18-27-PE pentamer: https://www.proimmune.com/introduction-to-pentamers/

Mouse CD45 (BD Biosciences):https://www.bdbiosciences.com/us/reagents/research/antibodies-buffers/immunology-reagents/anti-mouse-antibodies/cell-surface-antigens/buv661-rat-anti-mouse-cd45-30-f11/p/612975

Mouse CD11b (BD Biosciences): https://www.bdbiosciences.com/us/applications/research/stem-cell-research/mesenchymal-stem-cell-markers-bone-marrow/mouse/negative-markers/buv395-rat-anti-cd11b-m170/p/563553

Mouse CD11c (BD Biosciences): https://www.bdbiosciences.com/us/reagents/research/antibodies-buffers/immunology-reagents/anti-mouse-antibodies/cell-surface-antigens/apc-cy7-hamster-anti-mouse-cd11c-hl3/p/561241

Mouse Ly6G (BD Biosciences): https://www.bdbiosciences.com/us/reagents/research/antibodies-buffers/immunology-reagents/anti-mouse-antibodies/cell-surface-antigens/buv737-rat-anti-mouse-ly-6g-1a8/p/741813

Mouse CD19 (BD Biosciences): https://www.bdbiosciences.com/us/reagents/research/antibodies-buffers/immunology-reagents/anti-mouse-antibodies/cell-surface-antigens/buv805-rat-anti-mouse-cd19-1d3/p/749027

Mouse F4/80 (Biolegend): https://www.biolegend.com/en-us/products/fitc-anti-mouse-f4-80-antibody-4067

Mouse CD4 (Biolegend): https://www.biolegend.com/en-us/products/brilliant-violet-421-anti-mouse-cd4-antibody-7349

Mouse CD8a (Biolegend): https://www.biolegend.com/en-us/products/percp-cyanine5-5-anti-mouse-cd8a-antibody-4255

Mouse CD44 (BD Biosciences): https://www.bdbiosciences.com/us/reagents/research/antibodies-buffers/immunology-reagents/anti-mouse-antibodies/buv496-rat-anti-mouse-cd44-im7/p/741057

Mouse CD62L (Biolegend): https://www.biolegend.com/en-us/products/brilliant-violet-650-anti-mouse-cd62l-antibody-17377

Mouse CD69 (BD Biosciences): https://www.bdbiosciences.com/us/reagents/research/antibodies-buffers/immunology-reagents/anti-human-antibodies/cell-surface-antigens/buv563-hamster-anti-mouse-cd69-h12f3/p/741234

Annexin A5 (Biolegend): https://www.biolegend.com/en-us/products/apcfire-750-annexin-v-18129

7-AAD (Biolegend): https://www.biolegend.com/en-us/products/7-aad-viability-staining-solution-1649

Eukaryotic cell lines

Policy information about cell lines

Cell line source(s)	Raji lymphoma (ATCC, Cat# CCL86), LNCaP C42 (provided by Michel Sadelain, MSKCC), HepG2-core (provided by Antonio Bertoletti; Duke-NUS Medical School), Eu-ALL01 (provided by Michel Sadelain, MSKCC). Plat-A retroviral packaging cell line (Cellbiolabs.com, Cat# RV-102).
Authentication	Raji lymphoma was directly ordered from ATCC. No additional cell authentication was performed. All cell lines provided by collaborators, have been extensively characterized in published reports (Davila ML, PLoS One. 2013 Ap 9;8(4):e61338. We therefore did not further authenticate these cells.
Mycoplasma contamination	These cell lines tested negative for Mycoplasma using the MycoAlertTM Mycoplasma Detection Kit (Lonza, Cat# LT07-118)
Commonly misidentified lines (See ICLAC register)	No commonly misidentified cells lines were used in this study.

Animals and other organisms

Policy information about studies involving animals; ARRIVE guidelines recommended for reporting animal research

Laboratory animals	-- Male (for prostate cancer study) and Female NOD.Cg-Prkdcscid Il2rgtm1Wjl/SzJ (NSG) mice, 4-6 weeks old, bred in-house. IACUC Assurance #A3226-01 (Fred Hutchinson Cancer Research Center)<br>-- Female 6-8-week old Sprague Dawley rats (Taconic Biosciences)<br>--Female 4-6 albino B6 (C57BL/6J-Tyr<c-2J>) mice (Jackson Laboratories)
Wild animals	The study did not involve wild animals.
Field-collected samples	The study did not involve samples collected from the field.
Ethics oversight	The care and use of mice in this study was approved by the Institutional Animal Care & Use Committee (IACUC) at the Fred Hutchinson Cancer Research Center, and was in compliance with all relevant ethical regulations for animal testing and research (Assurance #A3226-01, IACUC Protocol Number 50782).

Note that full information on the approval of the study protocol must also be provided in the manuscript.

Human research participants

Policy information about studies involving human research participants

Population characteristics	*Describe the covariate-relevant population characteristics of the human research participants (e.g. age, gender, genotypic information, past and current diagnosis and treatment categories). If you filled out the behavioural & social sciences study design questions and have nothing to add here, write "See above."*
Recruitment	*Describe how participants were recruited. Outline any potential self-selection bias or other biases that may be present and how these are likely to impact results.*
Ethics oversight	*Identify the organization(s) that approved the study protocol.*

Note that full information on the approval of the study protocol must also be provided in the manuscript.

Flow Cytometry

Plots

Confirm that:

☒ The axis labels state the marker and fluorochrome used (e.g. CD4-FITC).

☒ The axis scales are clearly visible. Include numbers along axes only for bottom left plot of group (a 'group' is an analysis of identical markers).

☒ All plots are contour plots with outliers or pseudocolor plots.

☒ A numerical value for number of cells or percentage (with statistics) is provided.

Methodology

Sample preparation	Tissues were harvested and minced into 2-4 mm pieces using scissors and digested using Collagenase/Hyaluronidase (Stemcell, Catalog #07912) before sieving the cell suspension through a 100 µm cell strainer. Prior to antibody labeling, the cell suspension was incubated at 4 °C for 10 min with anti-murine CD16/CD32 FC-Receptor blocking reagent (final concentration of 2.5 µg/ml, dilution factor 1:200) to prevent unspecific binding.
Instrument	Data were acquired using a BD FacsCanto II cell analyzer
Software	FACSDIVA software (acquisition), FlowJo v10.1 (analysis)
Cell population abundance	The cell population of interest (CAR T cells) were present in cell suspension at frequencies ranging from 0.1% to 40% (depending on the treatment group).
Gating strategy	Cells were first gated for singlets (FSC-H vs. FSC- A) this gate was further analyzed for uptake of the Live/Dead Aqua stain to determine live versus dead cells and their expression of CD8 and C-myc (CAR), taking only the live, healthy cell population.

☒ Tick this box to confirm that a figure exemplifying the gating strategy is provided in the Supplementary Information.
